# Super-Resolution Imaging with Ultrasound for Visualization of the Renal Microvasculature in Rats Before and After Renal Ischemia: A Pilot Study

**DOI:** 10.3390/diagnostics10110862

**Published:** 2020-10-22

**Authors:** Sofie Bech Andersen, Iman Taghavi, Carlos Armando Villagómez Hoyos, Stinne Byrholdt Søgaard, Fredrik Gran, Lars Lönn, Kristoffer Lindskov Hansen, Jørgen Arendt Jensen, Michael Bachmann Nielsen, Charlotte Mehlin Sørensen

**Affiliations:** 1 Department of Radiology, Rigshospitalet, 2100 Copenhagen, Denmark; stinne.byrholdt.soegaard@regionh.dk (S.B.S.); lars.birger.loenn@regionh.dk (L.L.); Kristoffer.Lindskov.Hansen.01@regionh.dk (K.L.H.); mbn@dadlnet.dk (M.B.N.); 2 Department of Biomedical Sciences, University of Copenhagen, 2200 Copenhagen, Denmark; cmehlin@sund.ku.dk; 3 Department of Clinical Medicine, University of Copenhagen, 2200 Copenhagen, Denmark; 4 Center for Fast Ultrasound Imaging, Department of Health Technology, Technical University of Denmark, 2800 Lyngby, Denmark; imat@dtu.dk (I.T.); jaje@dtu.dk (J.A.J.); 5 BK Medical ApS, 2730 Herlev, Denmark; choyos@bkmedical.com (C.A.V.H.); fgran@bkmedical.com (F.G.)

**Keywords:** super-resolution ultrasound imaging, rats, Sprague-Dawley, kidney, microcirculation, vasa recta, reperfusion injury

## Abstract

In vivo monitoring of the microvasculature is relevant since diseases such as diabetes, ischemia, or cancer cause microvascular impairment. Super-resolution ultrasound imaging allows in vivo examination of the microvasculature by detecting and tracking sparsely distributed intravascular microbubbles over a minute-long period. The ability to create detailed images of the renal vasculature of Sprague-Dawley rats using a modified clinical ultrasound platform was investigated in this study. Additionally, we hypothesized that early ischemic damage to the renal microcirculation could be visualized. After a baseline scan of the exposed kidney, 10 rats underwent clamping of the renal vein (*n* = 5) or artery (*n* = 5) for 45 min. The kidneys were rescanned at the onset of clamp release and after 60 min of reperfusion. Using a processing pipeline for tissue motion compensation and microbubble tracking, super-resolution images with a very high level of detail were constructed. Image filtration allowed further characterization of the vasculature by isolating specific vessels such as the ascending vasa recta with a 15–20 μm diameter. Using the super-resolution images alone, it was only possible for six assessors to consistently distinguish the healthy renal microvasculature from the microvasculature at the onset of vein clamp release. Future studies will aim at attaining quantitative estimations of alterations in the renal microvascular blood flow using super-resolution ultrasound imaging.

## 1. Introduction

In vivo visualization of the microvasculature is an important clinical tool. The microvascular networks are fundamental for tissue homeostasis and are altered by diseases such as diabetes, ischemic disease, or cancer as well as normal aging [1,2,3,4,5,6,7]. The architectural or functional alterations of the microvasculature can compromise organ function or facilitate tumor growth. Therefore, early and precise diagnosis and monitoring of the microvascular alterations are crucial to detect these unfavorable microcirculation effects. Super-resolution (SR) ultrasound imaging can depict the vasculature, including the microvasculature, of both superficial and deeper-lying organs and tissues in vivo using sparsely distributed microbubbles (MBs) as intravascular tracers. The SR image is obtained over minutes and is an accumulation of thousands of successive image frames in which each MB centroid is detected [8,9]. Furthermore, MB track maps can display estimations of MB velocity and direction based on tracking the MBs between frames. The healthy microvasculature of rodent organs and tissues has been visualized with SR imaging [8,9,10,11,12,13]. SR imaging can also visualize and quantify the pathological microvascular patterns in rodent and chicken embryo tumors [14,15,16] and the later stages of acute kidney injury in mice [17]. With a rich and anatomically distinctive vasculature [18], the kidneys are attractive organs for SR imaging. In rodents, ischemia-reperfusion can be used as a model of acute kidney injury, and contrast-enhanced ultrasound studies have shown that the insult causes prolonged reduction of the renal blood flow [19,20,21], probably due to impaired vascular reactivity and microvascular damage [22].

This study aimed to visualize the renal vasculature in male Sprague-Dawley rats using a modified commercial ultrasound scanner and probe. In addition, to gain initial clinical experience with this SR imaging setup, we investigated whether renal ischemia-reperfusion injuries could be identified using SR images acquired before, immediately after, and one hour after the insult. The SR results were compared with corresponding conventional power Doppler ultrasound scans. We hypothesized that the SR technique would allow us to visualize acute renal microvascular damage in vivo. We found that the SR technique revealed consistent and highly detailed depictions of the renal vascular tree, including the ascending vasa recta with a 15–20 μm diameter. The vasa recta were not visible with conventional power Doppler. The SR images alone did not consistently reveal renal blood flow changes after ischemia-reperfusion. Future studies will aim to quantify alterations in the renal microvascular blood flow using MB track maps.

## 2. Materials and Methods

### 2.1. Animal Ethics and Preparation

The experiments were performed in accordance with protocols approved by the Danish Animal Experiments Inspectorate under the Ministry of Environment and Food (license number 2015-15-0201-004637, issued on 17 April 2015). The study was performed at the University of Copenhagen, and all procedures agreed with the ethical standards of the university which comply with the EU Directive 2010/63/EU for animal experiments. The animal facility at the Department of Experimental Medicine, University of Copenhagen housed the rats, and animal caretakers were responsible for their wellbeing until use. The rats were held in a 12/12-h light/dark cycle and had free access to water and standard chow. The study was conducted on a total of 15 healthy male Sprague-Dawley rats (mean weight: 322 g, standard deviation (SD) ± 49 g) obtained from Taconic A/S (Lille Skensved, Denmark). Ten rats underwent ischemia-reperfusion with three consecutive SR scans. Another three rats underwent the same SR protocol as the 10 rats but without ischemia-reperfusion as sham controls for histological evaluation to compensate for the three periods with MB infusion. One rat had an SR scan followed by an ex vivo magnetic resonance imaging (MRI) scan. The last rat was included from a pre-trial to show a different imaging plane. Anesthesia was induced with 5% isoflurane in 65% nitrogen and 35% oxygen; 1–2% isoflurane maintained the anesthesia. Ventilation was ensured by tracheotomy with connection to a mechanical ventilator (Ugo Basile, Gemonio, Italy) with 72 respirations/min. Two polyethylene catheters (PE-10) were inserted in the right jugular vein and used for the infusion of MBs (SonoVue, Bracco Imaging, Milan, Italy) and isotonic saline mixed with Nimbex (cisatracurium, 0.85 mg/mL, GlaxoSmithKline, London, UK, 20 µL/min). The mean arterial pressure (MAP) was measured using a polyethylene catheter (PE-50) in the left carotid artery and a Statham P23-dB pressure transducer (Gould, Oxnard, CA, USA). The rats were placed in the supine position on a heating table to ensure a steady body temperature (37 °C), and the left kidney was exposed through laparotomy. To further expose the kidney and reduce respiratory motion, a metal retractor pulled the left side of the diaphragm slightly in the cranial direction. Rat data are available in the Supplementary Materials, Table S1.

### 2.2. Ultrasound Scanning, Ischemia-Reperfusion Procedure, and Histopathology

**Figure 1:**
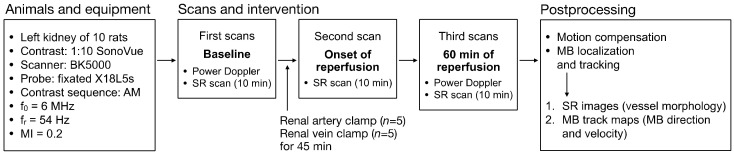
Experiment overview of the ischemia-reperfusion intervention. AM = amplitude modulation, f_0_ = center frequency of the probe, f_r_ = frame rate, MI = mechanical index, SR = super-resolution, MB = microbubble.

An overview of the ischemia-reperfusion experiments is illustrated in Figure 1.

Imaging data were obtained using a bk5000 scanner and a fixated X18L5s transducer (BK Medical ApS, Herlev, Denmark). The scanner was modified with a research interface for the live streaming of beamformed radio-frequency data to a disk. The transducer was placed on the lateral surface of the left kidney with gel for coupling. B-mode imaging was used to optimize the imaging plane, making sure both cortical and medullary regions were represented. The MBs were diluted in isotonic saline (1:10) and injected at a rate of 100 µL/min. The MB dilution was made anew before each scan to ensure MB integrity. Data recording was initiated when MBs became visible on the contrast-enhancing sequence display, and the SR data were acquired over 10 min [23]. To ensure a continuous influx of MBs, a custom-built device turned the syringe 180 degrees every 60 s [24]. After a baseline SR scan, five rats had their renal artery clamped, and five rats had their renal vein clamped using a nontraumatic micro-clamp. Both renal vein and artery clamping were included to investigate different severities of tissue damage [25,26]. After 45 min, the clamp was removed and a second SR scan was performed at the onset of reperfusion. The last SR scan was performed in a steadier state, 60 min after clamp release. In between the scans, the kidney was superfused with heated saline (37 °C). Before the second and third SR scan, the imaging plane was readjusted with reference to the baseline B-mode image. For comparison, power Doppler ultrasound (PRF: 400 Hz, wall filter cutoff: 80 Hz, center frequency for transmission: 9.5 MHz) was acquired in direct continuation of the baseline and third SR scan, using the same scanner and transducer. All the rats were euthanized in anesthesia immediately after the experiments. The left kidney from the 10 rats that underwent ischemia-reperfusion and both kidneys from the three sham rats were removed and fixated in 4% paraformaldehyde (PFA) for documentation of the tissue damage. The kidneys were embedded in paraffin, cut into 4 µm slices, and stained using a standard hematoxylin and eosin (H&E) protocol. The images were analyzed by a trained anatomist blinded to the intervention category.

### 2.3. SR Imaging Technique and Data Processing

Contrast images were created using an amplitude modulation sequence (half-power, full-power, half-power) interleaved with B-mode images. Data were acquired using line-per-line focused beam transmission (frame rate: 54 Hz, center frequency for transmission: 6 MHz, mechanical index: 0.2). Off-line processing was conducted using an SR imaging pipeline programmed in MATLAB at the Technical University of Denmark. Nonrigid motion of the kidney was estimated by tissue speckle tracking [27]. The motion was compensated by using the motion estimates to adjust the individual MBs back to their location on the reference image. MBs were localized using thresholding and centroid detection, and tracks were made by connecting the nearest MBs in consecutive frames. Afterward, the tracks were inserted in the high-resolution images to yield SR images and the MB velocities estimated from the tracks were used to create the MB track maps. A color wheel indicated the MB direction by color and color brightness was proportional to velocity. To highlight specific parts of the vasculature, MB trajectories were classified and shown based on their length, flow direction, velocity, and lifetime index (the number of consecutive frames an MB was tracked). An increase in track length and MB lifetime removed false tracks, leaving the most robust tracks in the images.

### 2.4. Evaluation of SR Images and Blood Pressure Data

The SR and power Doppler images from the 10 rats that underwent ischemia-reperfusion were independently and blindly evaluated by six of the co-authors. The assessors had different backgrounds qualifying them for SR image assessment: C.M.S. is associate professor in human biology with expertise in renal physiology, M.B.N. is professor in radiology with expertise in vascular ultrasound, L.L. is professor in radiology with expertise in vascular interventional radiology, K.L.H. is a radiologist with expertise in vascular ultrasound, C.A.H.V. is an ultrasound engineer with expertise in vascular ultrasound, and S.B.S. is an M.D. working with SR imaging in a PhD. All assessors had basic knowledge about the SR technique (experience ranging from 6 months to 5 years of working with SR imaging), as well as knowledge about rat kidney vascular anatomy and received written instructions prior to completing the evaluation. The written instructions for each assignment are shown in the Supplementary Materials, Tables S2, S4–S6. The three SR images from each rat were categorized as either baseline (the 10 images the assessors believed look normal based on their basic knowledge about SR imaging and renal vascular anatomy), onset of reperfusion (the 10 images that fit an assumption of low renal blood flow at the onset of reperfusion after a longer period of ischemia) or 60 min of reperfusion (the 10 images that match the assumption that the renal blood flow is restored to some degree). The power Doppler images from each rat were categorized as baseline or 60 min of reperfusion. After this, all scans were categorized as artery or vein clamp intervention, knowing that vein clamping induces most tissue damage [25,26]. The results were calculated as percentages of correct answers from all assessors. Interobserver agreement was tested with a fixed-marginal multi-rater kappa (using the Online Kappa Calculator, available online: http://justusrandolph.net/kappa/ (accessed on 27 May 2020)). Differences in blood pressure during the scans were tested with a mixed-effects model (REML) with repeated measures with a Greenhouse-Geisser correction using GraphPad Prism 8.0 (version 8.4.3 for mac, GraphPad Software, San Diego, CA, USA).

### 2.5. Ex Vivo MRI of the Microvasculature

To compare the SR images of the healthy renal microvasculature with another imaging modality, ex vivo MRI was made of a single specimen. The rat was anesthetized and tracheotomized as described above. A polyethylene catheter (PE-25) was placed at the left renal artery through the femoral artery, and the aorta cranially to the left renal artery as well as the right renal artery were closed with sutures. Additional sutures were placed at the left renal artery, left renal vein, and left ureter. The rat was euthanized, and the kidney was perfused with 3 mL of 4% PFA followed by 2 mL containing 1 mL 1:10 diluted GadoSpin P (Viscover, Miltenyi Biotec, Bergisch Gladbach, Germany) and 1 mL 4% PFA. When the injection was completed, the remaining sutures were closed, and the specimen was immersed in 4% PFA. The kidney was scanned for 17 h in a 9.4T preclinical MR scanner (BioSpec 94/30 USR, Bruker BioSpin, Ettlingen, Germany) with a T1 contrast-enhanced 3D spoiled gradient echo sequence (3D Fast Low Angle Shot: 3D-FLASH sequence) [28]. Images were acquired using a 1H cryogenically cooled quadrature-resonator Tx/Rx coil (CryoProbe, Bruker BioSpin). MR image voxel size was 30 µm isometric. To get a comparable representation of the vasculature, the corresponding 2.5 mm maximum intensity projection MR image slice was reconstructed and compared with the SR images (slice thickness ~0.6–1.2 mm).

## 3. Results

### 3.1. The Healthy Renal Microvasculature

**Figure 2:**
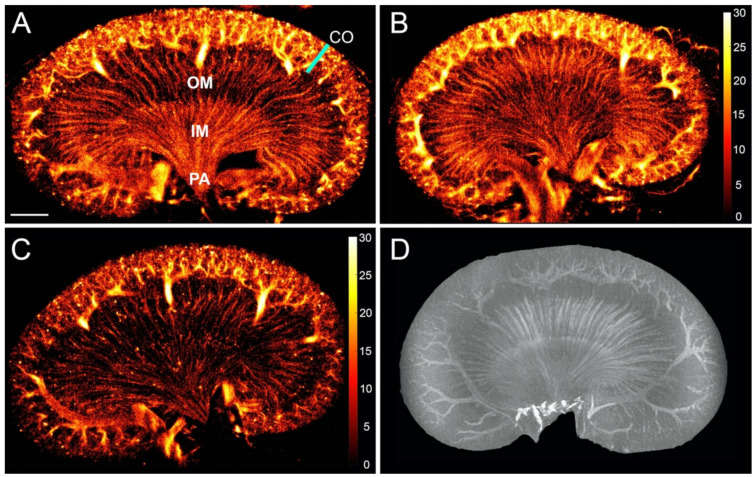
(**A**–**C**) Super-resolution images (log-scaled, i.e., color bar shows the value of intensity after logarithmic compression, ranging from 0~30 dB, and intensity corresponds to the number of microbubbles) of the healthy rat renal microvasculature. (**A**) There is a clear distinction between the dense cortical (CO) microvascular network and the vasa recta of the outer medulla (OM) and inner medulla (IM), leading down to the papilla (PA). Even though the scans were performed under the same conditions, the images show how the number of microbubbles varied between the rats, indicated by the higher intensity on image (**B**) (average of 117 detected microbubbles/frame) compared with image (**C**) (average of 70 detected microbubbles/frame). (**D**) Ex vivo magnetic resonance T1 contrast-enhanced image of another rat kidney for comparison. The kidneys measure approximately 2 cm in craniocaudal length and 1 cm in the medial-lateral direction. Scale bar: 2 mm.

In the healthy rat kidneys, a mean of 74 ± 27 MBs/frame was detected during the scans (32,300 frames). The SR images displayed the characteristic anatomical structure of the renal vasculature in the unipapillar rat kidney with the dense cortical vasculature clearly distinguishable from the vasa recta of the medulla (Figure 2A–C). The variations in the images could be caused by a combination of the normal variation in renal artery branching and renal morphology [29] as well as small differences in the imaging plane. The renal vascular structure found with ultrasound SR imaging was comparable to that found with ex vivo MRI (Figure 2D).

**Figure 3:**
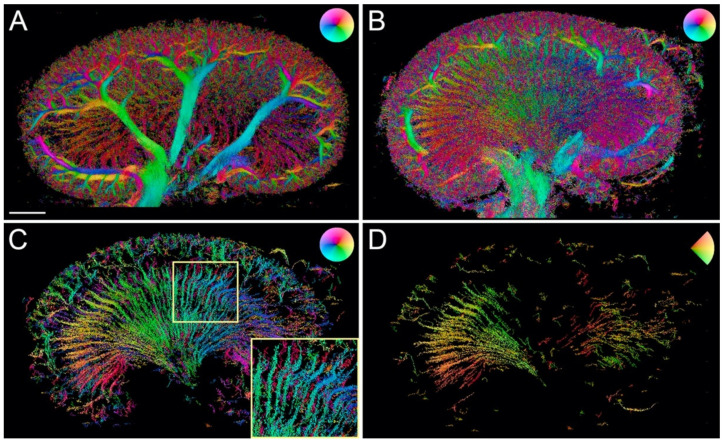
Super-resolution microbubble track maps. (**A**) Unfiltered microbubble track map. The color of the wheel indicates microbubble flow direction, and the color brightness indicates MB flow velocity. The image shows the opposite flow of the paired arteries and veins of the renal vascular tree. (**B**) Without filtering, this velocity map (from another rat) contains a high number of tracks (343,939). (**C**) The microbubble track map from (**B**) filtered to increase the robustness of the included tracks and highlight the long straight vasa recta, thereby allowing a distinction between the descending (green/blue) and ascending (red) vasa recta (insert). (**D**) Another way to filter these maps is by direction. This image is filtered to show microbubbles with a direction going from left to right, leaving the descending vasa recta visible on the left side of the medulla, and the ascending visible on the right side. Scale bar: 2 mm.

The number of tracks in the MB track maps (Figure 3) ranged from 95,008 to 343,989. These maps allowed further delimitation and characterization of the renal vessels. Using the color wheel, the opposing flow directions in adjacent tracks helped discern arteries from veins (Figure 3A, included tracks with velocities from 0–10 mm/s). Without further image filtering, the MB track maps were difficult to read (Figure 3B, included tracks with velocities from 0–10 mm/s). In Figure 3C, the image from Figure 3B was filtered to include tracks that were longer than 300 μm, with an MB lifetime over 20 frames and a max velocity of 3 mm/s. This filtering highlighted the long straight vasa recta by removing some of the false or short tracks and enabled separation of the descending and ascending vasa recta. Figure 3D was filtered according to the direction and included only flow going from left to right, thereby highlighting the descending vasa recta in the left side and the ascending vasa recta in the right side of the medulla (other filtration parameters: tracks length > 250 μm, MB lifetime > 30 frames, max velocity 3 mm/s).

### 3.2. The Renal Microvasculature after Ischemia with Reperfusion

**Figure 4:**
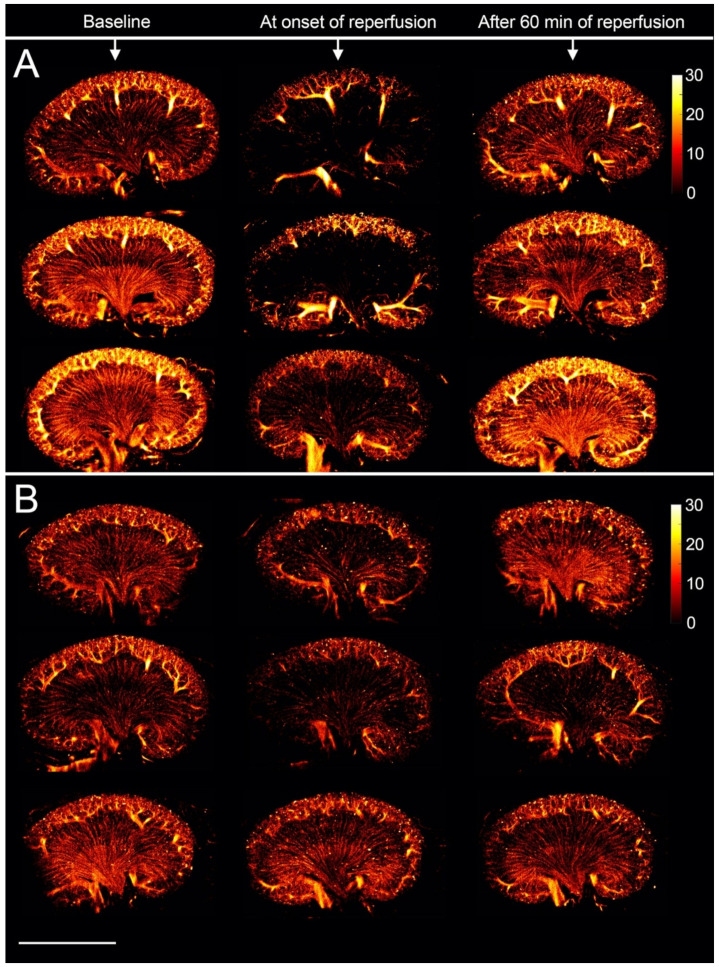
(**A**) Super-resolution images (log-scaled, i.e., color bar shows the value of intensity after logarithmic compression, ranging from 0~30 dB, and intensity corresponds to the number of microbubbles) of three rat kidneys before and after clamping of the renal vein. (**B**) Super-resolution images of three rat kidneys before and after clamping of the renal artery. Scale bar: 10 mm.

At the onset of reperfusion after release of the vein clamp, the SR images depicted the entire renal vasculature poorly perfused with MBs compared with baseline. It was particularly noticeable for the vasa recta, as exemplified in Figure 4A (middle section). The results were subtler at the onset of reperfusion after renal artery clamping where some of the kidneys did not alter from the baseline (Figure 4B, middle section). After 60 min of reperfusion, MBs refilled the microvascular bed in varying degree among the 10 rats. The vasculature appeared more irregular in some samples, while others showed a complete recovery of the microvascular flow after the intervention. All SR images of the 10 rats are available in the Supplementary Materials in Figures S1 and S2.

**Figure 5:**
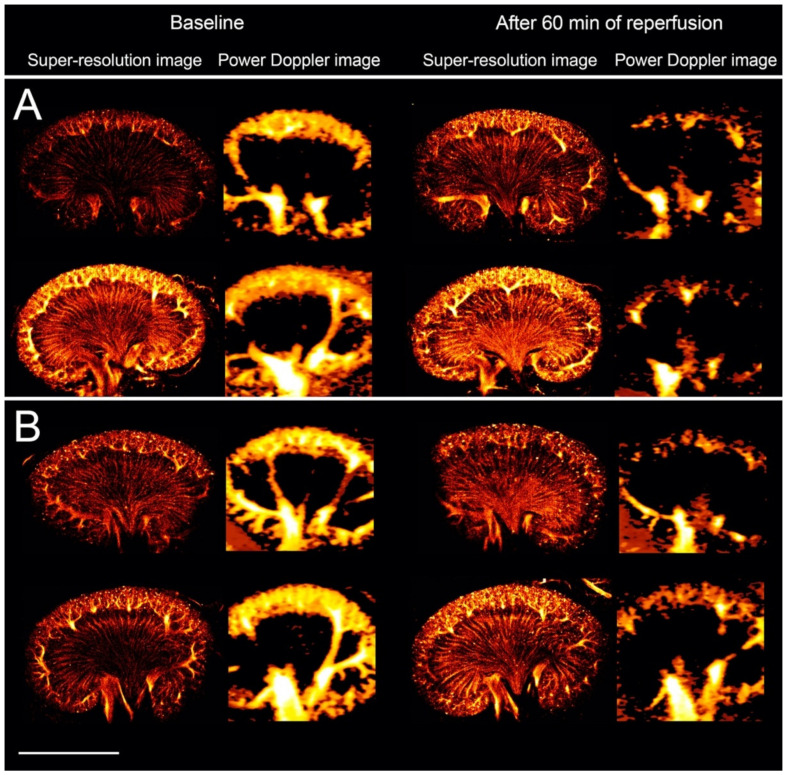
(**A**) Super-resolution (log-scaled) and power Doppler images of two rat kidneys before and after clamping of the renal vein. (**B**) Super-resolution (log-scaled) and power Doppler images of two rat kidneys before and after clamping of the renal artery. In these examples, all the super-resolution images showed a complete refilling of the microvascular bed after 60 min of reperfusion. The power Doppler scans from all animals showed a decreased signal in the cortex after 60 min of reperfusion compared with baseline. Scale bar: 10 mm.

Six blinded assessors correctly identified the five scans as the onset of reperfusion after vein clamping, while the percentage of correctly identified scans at the onset of reperfusion after artery clamping was only 60%. The assessors were not able to distinguish the baseline scans from the scans after 60 min of reperfusion. For the vein clamping, 63% were correctly identified as baseline or 60 min of reperfusion. For the artery clamping, 40% were correctly identified as baseline and 30% as 60 min of reperfusion. The fixed-marginal kappa was 0.60, showing only moderate agreement among the assessors. When evaluating all the SR scans in the correct order, the assessors correctly identified 67% as either vein or artery clamping. The power Doppler scans visualized only the cortical blood flow. After 60 min of reperfusion, all the power Doppler images showed a decrease in the cortical blood flow compared with baseline (exemplified in Figure 5). The assessors correctly identified all of the 20 power Doppler scans as either baseline or 60 min of reperfusion, and correctly identified 80% as either vein or artery clamping. All power Doppler images as well as the image assessment results are available in the Supplementary Materials in Figures S3 and S4 and Tables S2–S6, respectively.

There was no statistically significant difference in the MAP measured during the three scans (*p* = 0.21). During the baseline scans, the mean of the MAP for the 10 rats was 109 ± 19 mmHg, and at the onset of reperfusion it was 112 ± 22 mmHg. At 60 min of reperfusion, one rat had missing data, and the MAP was 106 ± 23 mmHg.

### 3.3. Histopathological Evaluation

**Figure 6:**
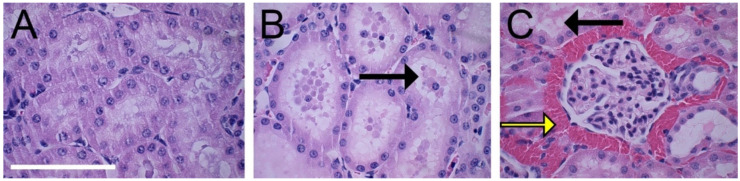
Hematoxylin and eosin staining of the renal cortex from three rats. (**A**) Control. (**B**) After renal artery clamping. (**C**) After renal vein clamping. The black arrows show intratubular cast formation in the tubules. The yellow arrow shows erythrocytes accumulated in the renal interstitial space around a glomerulus. Scale bar: 100 μm.

Examples of H&E stains from three rats are shown in Figure 6. The five vein clamp specimens were all correctly identified. They revealed a high number of erythrocytes accumulated in the renal interstitial space. Some of the specimens, both after artery and vein clamping, showed intratubular cast formation as a sign of ischemic damage. However, the assessor could not correctly identify all the arterial clamp (60% correctly identified) and sham kidney specimens (67% correctly identified). The results from histopathological evaluation are available in the Supplementary Materials, Table S7.

## 4. Discussion

We investigated the ability to create SR images of the renal vasculature of healthy Sprague-Dawley rats using a modified clinical ultrasound scanner and probe together with a customized processing pipeline for tissue motion compensation and MB tracking. The SR images revealed a consistent and detailed depiction of the renal vascular tree, including the microvasculature, similar to descriptions from anatomical studies [18,29], to ex vivo nano-CT images of mouse kidneys [30], and to the vascular structure of a rat kidney found with ex vivo MRI. MBs were tracked in the ascending vasa recta with a diameter of approximately 15–20 μm [31,32]. The descending vasa recta are clustered in vascular bundles and were therefore also visible. Since the sulfur hexafluoride gas in SonoVue is exhaled and MBs act similarly to erythrocytes, the SR images visualize only the renal vasculature and not the tubular system [33,34]. To our knowledge, the presented images reveal the highest level of detail of the renal vascular tree of rats obtained with modified clinical ultrasound platforms. Some studies using ultrasound SR imaging on rodent kidneys have not visualized the vasa recta, which could be due to short scan times or out-of-plane motion [17,35]. SR imaging for visualization of the slowly flowing blood in the vasa recta requires data acquisition to be lengthy to fill as many of the small vessels with MBs as possible for an adequate microvascular representation [23]. As mentioned, the variation in the images of the healthy kidneys may be due to known variations in the renal artery branching and renal morphology [29]. Regarding the microvasculature, it is still not completely clarified how many times the renal cortical radiate arteries branch, and how many afferent arterioles branch from each subsegment of the cortical radiate arteries. Differences in the imaging plane and the detected number of MBs also cause image variations. Hence, these normal variations need to be considered in future studies where different groups of animals and eventually humans with a more complicated multipapillary renal anatomy are investigated and compared.

To gain initial experience with the technique in a clinical setting, the early vascular changes induced by renal ischemia-reperfusion were investigated with SR imaging and compared with power Doppler imaging. Ischemia-reperfusion is known to cause prolonged reductions in renal blood flow [19,20,21,22,36,37,38]. We scanned the kidneys intraabdominally to simplify the SR data by reducing motion from respiration and neighboring bowels that push the kidney out of the imaging plane. This setup confined the study to the very early stages of reperfusion. At the onset of reperfusion after obstructing the renal vein, the SR images showed a more notable reduction in the vascular refilling compared with the renal artery, as demonstrated by the image assessment and as expected, since vein occlusion causes more tissue damage [25,26]. After 60 min of reperfusion, the SR images revealed a varying degree of microvascular MB refilling, while the power Doppler scans showed reduced cortical blood flow in all rats. Power Doppler imaging is used in the clinic for visualization of blood flow but is limited in its ability to detect slow flow. Theoretically, the lowest detectable velocity for the power Doppler in this study was approximately 6.5 mm/s. Therefore, the vasa recta with a blood flow velocity around 1 mm/s were not visualized [10,32]. On the other hand, the velocity cut off for power Doppler enabled the visualization of the decrease in cortical blood flow. The SR images are accumulations of tracks from the 10 min recording, each track with a specific velocity. Therefore, a decrease in the blood velocity was not visually observable, as indicated by the image assessment. A study that used conventional contrast-enhanced ultrasound to investigate the early response to renal ischemia-reperfusion in mice also found variation in the response to the intervention [20]. Whether this initial degree of MB refilling of the vascular bed influences the outcome after the insult is important to explore [39,40]. Even though the results from this initial pilot study are not ideal yet, they serve as a platform for future experiments. Future work will aim at attaining transcutaneous kidney scans for longitudinal studies with this SR technique. This will allow us to investigate whether SR imaging is able to detect alterations in the microvascular architecture at the early stages of their occurrence. If early or subtle microvascular alterations have a prognostic value and could allow timely initiation or change of treatment, e.g., in persons who are developing diabetic nephropathy but do not yet show other clinical signs of disease [41], or in persons undergoing cancer treatment where early readouts of drug or radiation efficacy are crucial [42], ultrasound SR imaging could fill an unmet need and evolve into a strong tool in the clinical setting.

Other ultrasound techniques and imaging modalities are competing with the SR imaging setup presented in this paper. Ultrasound plane-wave techniques are commonly used to obtain SR images at a higher framerate [8,10,11,12,13,14,15,16,17]. To facilitate a faster implementation of SR imaging in the clinic, we used focused beam transmission which is an established technology in commercial ultrasound platforms. MRI and CT are also clinically relevant modalities for microvascular imaging of deeper-lying structures. We included an ex vivo MR image in this study. Since there are major differences between the in vivo SR imaging and ex vivo MRI, the MR image was included only as a simple reference for the SR images. This could also have been attained with ex vivo µCT [3,30,43,44]. The downsides of ex vivo imaging are the hour-long scan times, functional investigations are not possible, and the tissue fixation process can shrink the microvascular structures [45,46]. In addition, the number of contrast-filled vessels of the MRI specimen was lower than expected. This inadequate contrast-filling of the vessels is another challenge in ex vivo imaging [44]. In vivo MRI and µCT can provide maps of tissue oxygenation or perfusion and a relatively high-resolution when applied in small animal research [3,47,48,49], but also require long scan times and for µCT a high X-ray exposure. Human clinical scanners have even lower resolution. An advantage of ultrasound SR imaging is the possibility of direct human translation. The current setup with the hockey-stick transducer can be used on humans to achieve images of the same quality as presented here, given the out-of-plane motion is not excessive. The images will be restricted to a relatively small area given the probe’s aperture. A target could be superficial lymph nodes. Furthermore, the current scanner settings with a mechanical index of 0.2 and a center frequency of 6 MHz together with dilution of the MBs point to a low risk of adverse bio-effects [50]. If imaging larger organs that span deeper, another probe would be needed, and issues related to, e.g., appropriately high frame rate for MB tracking and differences in MB excitation in shallow vs deeper parts of the organ due to tissue attenuation would have to be addressed. Moreover, acoustic shadowing, associated with massively calcified vessels or kidney stones, is a common problem in ultrasound imaging. Since the performance of SR imaging relies on the echo signals from the MBs, extensive arteriosclerosis may affect MB detection and tracking in the vessels. Accordingly, the efficacy of SR imaging should be addressed when the technique is applied in animals or persons with chronic vascular disease. Nonetheless, compared with MRI and CT, the minutes spent attaining ultrasound images of deeper-lying microvascular structures should be acceptable when applied to the appropriate clinical settings.

This study was limited by the small number of animals. Another limiting factor is the lack of quantitative estimations of microvascular blood flow alterations during reperfusion. In chronic kidney disease the architecture of the vessels changes, resulting in reduced vessel density, decreased branching, or increased tortuosity [2,3,17]. The image assessment of the SR images in this study illustrated how the microvascular flow changes in an acute setup were not detectable by visual image evaluation. Moreover, visual image assessments are often affected by assessor subjectivity, further strengthening the need for quantitative image analysis strategies. However, to the best of our knowledge, a well-established way of flow quantification at this scale is still a big challenge. The MB number and behavior vary due to differences in anesthesia concentration, blood pressure, body weight, inflammation, and small variances in the handling of the MBs before injection [51], which was confirmed by the range in MB count in the baseline scans. Therefore, the number of MBs as a measure for perfusion is ambiguous, as also indicated by the image assessment. Estimations of MB velocity could reveal microvascular flow alterations. The renal vasculature is densely packed, and the MB track maps are composed of many thousand tracks, each with a unique direction, MB velocity, track length, and lifetime depending on anatomical region and vessel type. In addition, some vascular structures, such as the winding glomerular capillaries, are not possible to visualize with the current SR imaging techniques. Even though filtering allows isolation of specific vessel tracks, estimation of changes in velocities ranging from 1–2 mm/s are affected by even small uncertainties in the location of the MBs. Additionally, the MB tracking in this study was challenged by the relatively low frame rate of 54 Hz. Hence, the complexity of the track information makes quantification of microvascular flow changes an ongoing challenge. We anticipate that microvascular flow changes in the straighter arterioles e.g., the afferent arterioles or the vasa recta will be obtainable with SR imaging in the future. This will give important indirect insight into glomerular blood flow. Another limitation is the differences in the image planes due to probe re-adjustment prior to each scan. When comparing the three scans, anatomical or pathological information outside the imaging plane can be missed or misinterpreted. Studies with 3D reconstructions of 2D ultrasound techniques including SR imaging have been performed in animals including in rodent kidneys, rabbit lymph nodes and different rodent tumors [12,13,15,52,53]. The 3D reconstructions give a more complete morphological examination, but do not allow MB tracking in the elevational plane or motion compensation in all three directions. This could become feasible with other types of probes, e.g., row-column and matrix ultrasound probes that are currently being developed for in vivo SR imaging [54,55,56].

In conclusion, our study demonstrated that highly detailed ultrasound SR images of the healthy renal vasculature of rat kidneys are obtainable with modified clinical ultrasound equipment. The high level of anatomical detail of both the cortical and medullary vasculature suggests that the method can be applied in the investigation of a broad range of renal diseases. Future studies will aim at achieving quantitative estimations of the renal microvascular blood flow from the MB track maps as well as investigating diseases that disrupt the microvascular architecture to demonstrate the clinical usefulness of the technique.

## 5. Patents

Patent on the tissue motion correction algorithm by J.A.J. and I.T. used in this study has been purchased by BK Medical ApS, Herlev, Denmark.

## Supplementary Materials

The following are available online at https://www.mdpi.com/2075-4418/10/11/862/s1, Figure S1: Super-resolution images before and after renal artery clamping, Figure S2: Super-resolution images before and after renal vein clamping, Figure S3: Power Doppler images before and after renal artery clamping, Figure S4: Power Doppler images before and after renal vein clamping, Table S1: Experimental data, Table S2: Image assessment, assignment 1A, Table S3: Assignment 1A, interobserver agreement, Table S4: Image assessment, assignment 1B; Table S5: Image assessment, assignment 2A, Table S6: Image assessment, assignment 2B, Table S7: Assessment of hematoxylin and eosin staining.
