# Supplementary material for: Super-Resolution Imaging with Ultrasound for Visualization of the Renal Microvasculature in Rats Before and After Renal Ischemia: A Pilot Study
Source: Diagnostics (Basel). 2020 Oct 22;10(11):862. doi: 10.3390/diagnostics10110862 (PMC7690607; doi:10.3390/diagnostics10110862)
Supplement: Supplementary file 1 [file diagnostics-10-00862-s001.pdf]

Supplementary material

Super-Resolution Imaging with Ultrasound for Visualization of the Renal Microvasculature in Rats Before and After Renal Ischemia: A Pilot Study

Content

Pages 1-4 Super-resolution and power Doppler images of 10 rats.
Page 5 Experimental data (rat weight, blood pressure, track number and microbubble count).
Page 6-10 Image assessment. Assignment descriptions and answers from assessors.
Page 11 Histology assessment results.

1. Supplementary Figures

Super-resolution images before and after renal artery clamping

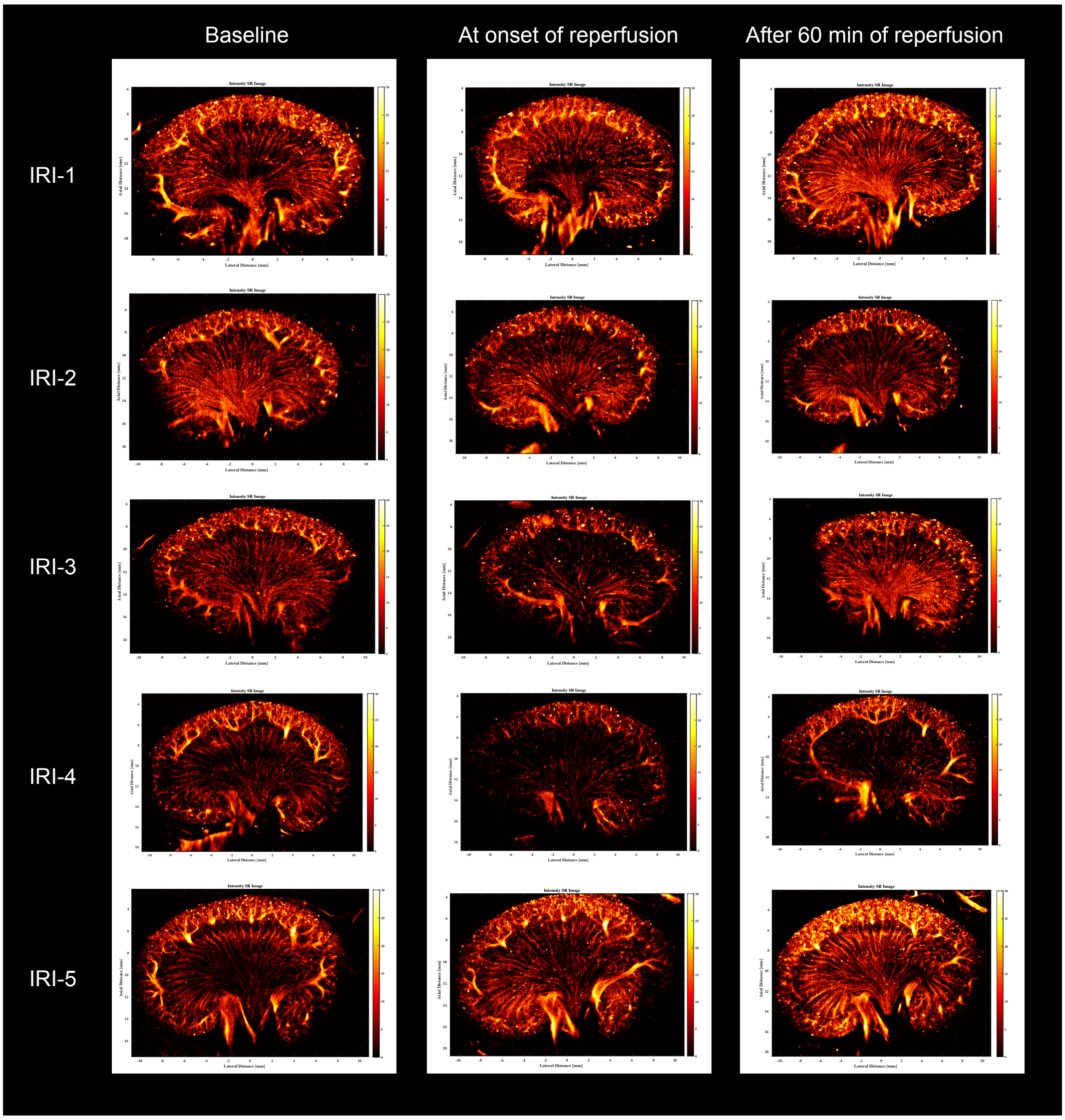

Figure S1. Super-resolution images of five rats before and after clamping the renal artery.

Super-resolution images before and after renal vein clamping

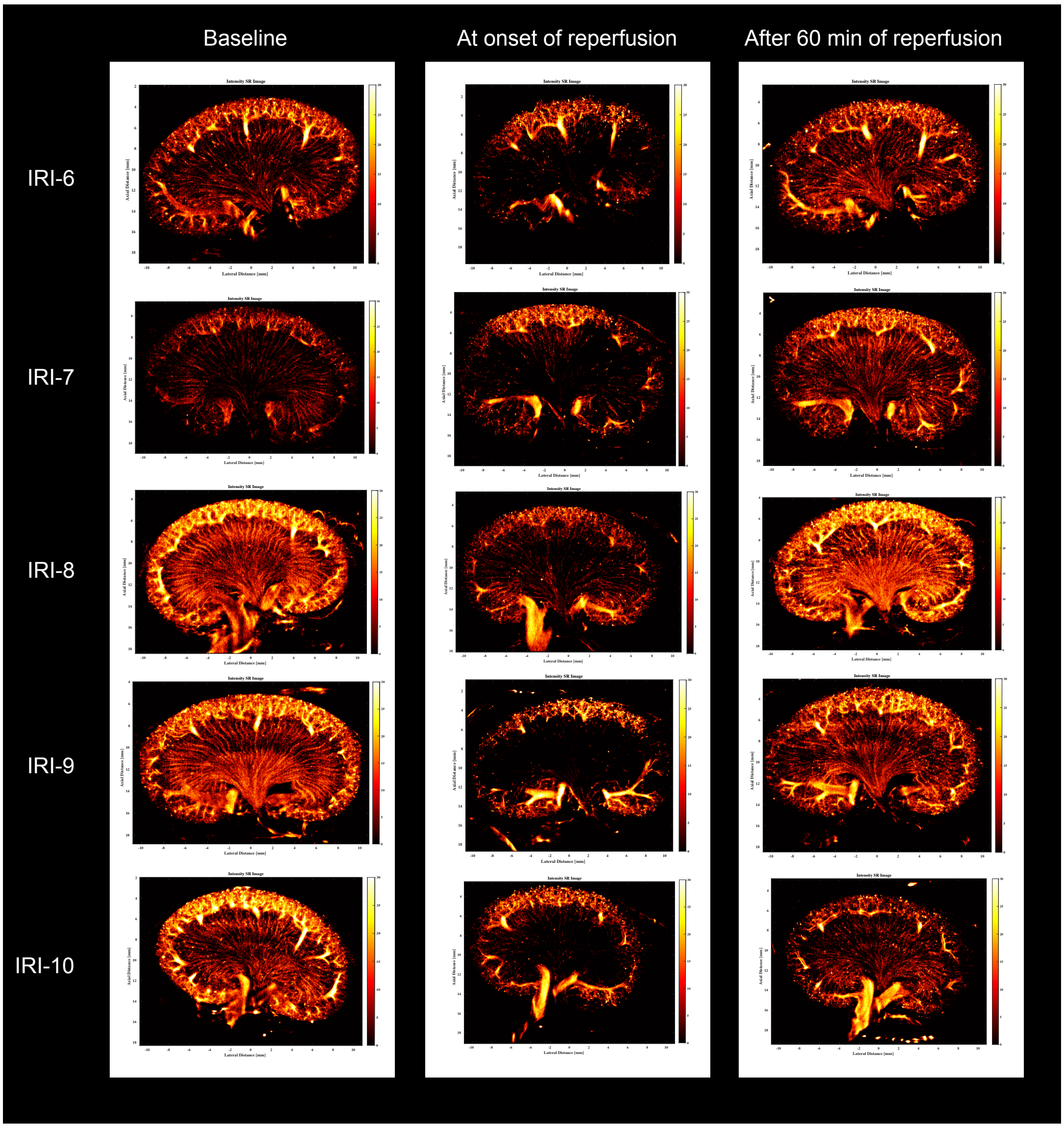

Figure S2. Super-resolution images of five rats before and after clamping the renal vein.

Power Doppler images before and after renal artery clamping

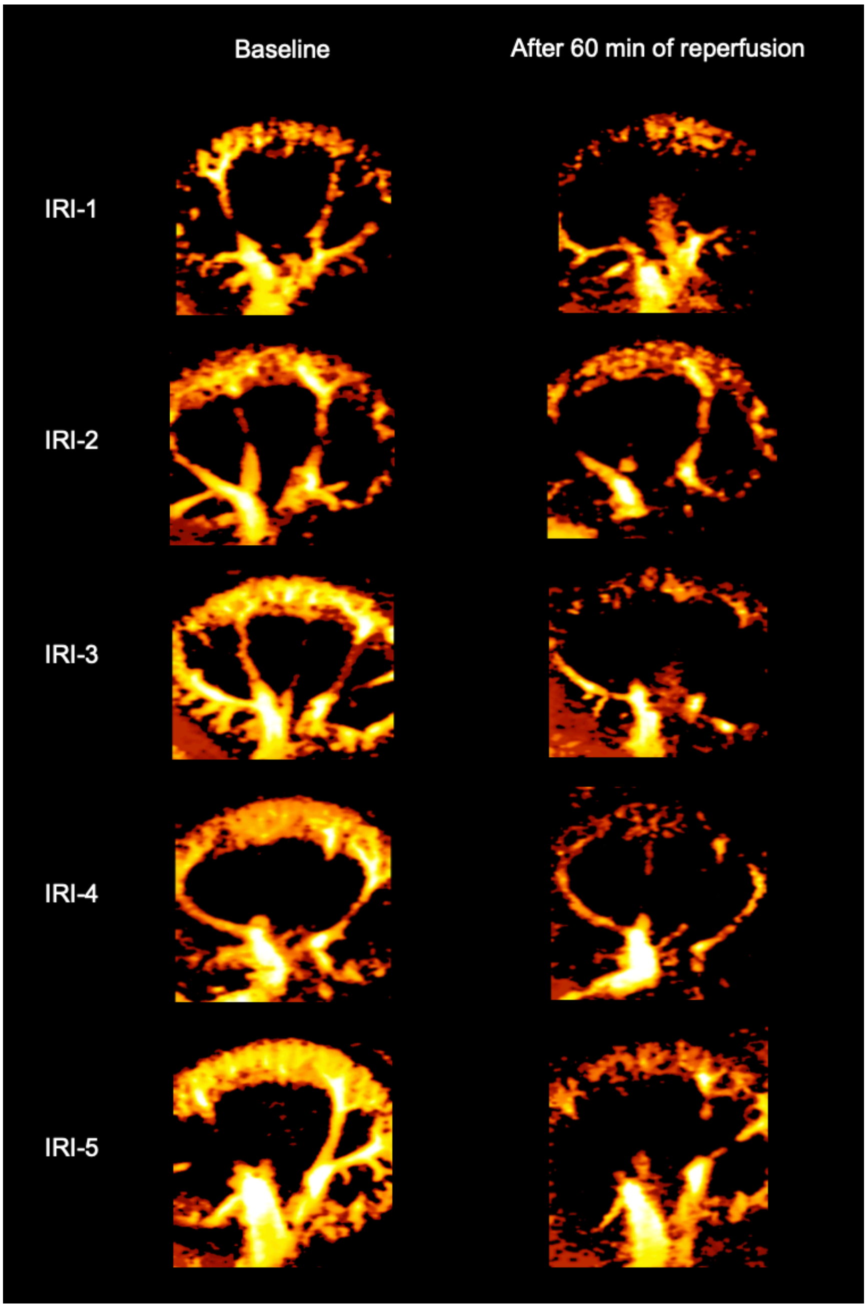

Figure S3. Power Doppler images of the five rats that had the renal artery clamped.

Power Doppler images before and after renal vein clamping

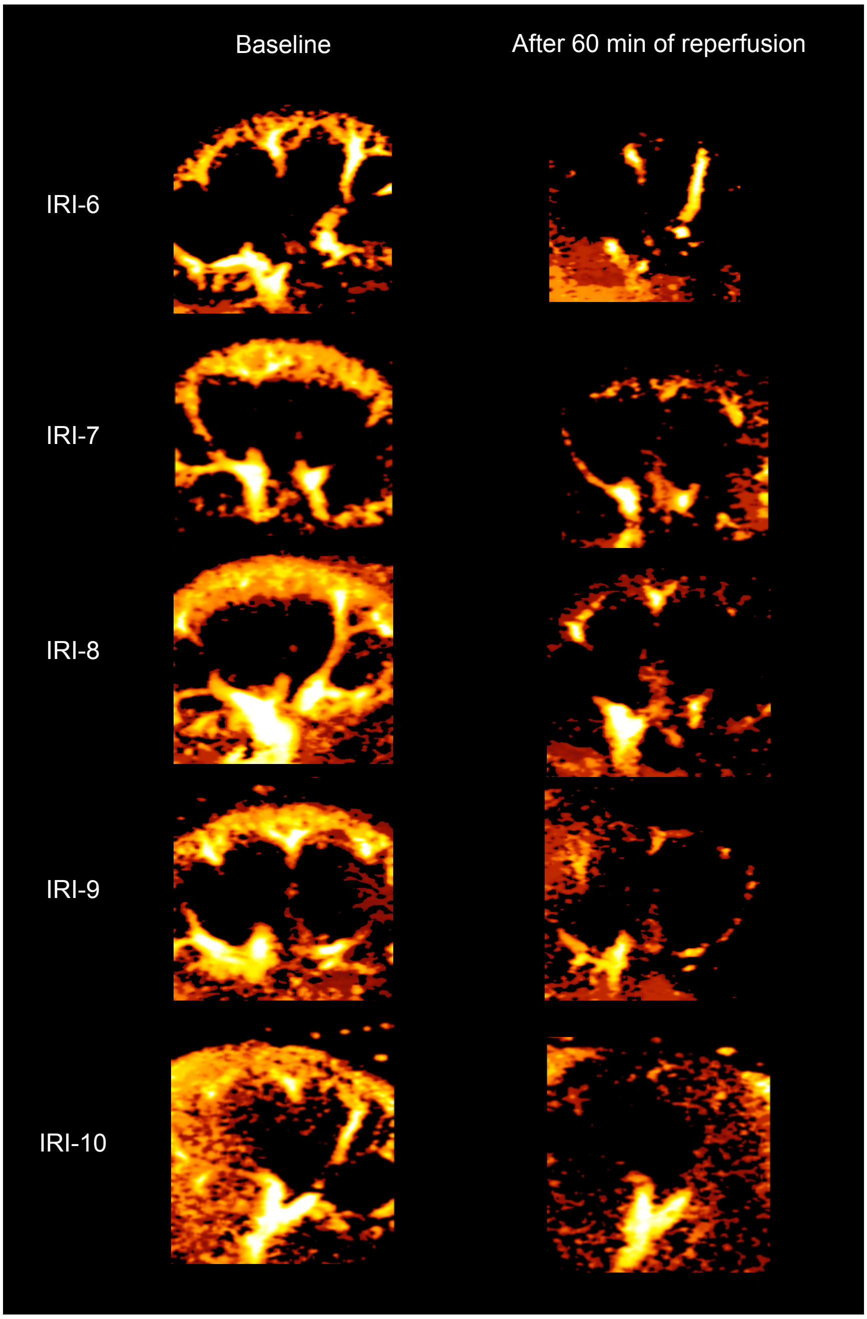

Figure S4. Power Doppler images of the five rats that had the renal vein clamped.

2. Supplementary Data

Table S1. Experimental data

			Mean Arterial Blood Pressure (mmHg) Average during the 10 min scan			Number of tracks (total)			Number of microbubbles (per frame)		
**Animal ID**	**Intervention**	**Weight (g)**	Baseline	Onset reperfusion	60 min reperfusion	Baseline	Onset reperfusion	60 min reperfusion	Baseline	Onset reperfusion	60 min reperfusion
IRI-1#	artery clamp	343	139.61	145.92	138.20	204,594	223,941	287,546	65	73	89
IRI-2	artery clamp	309	106.59	102.77	86.08	181,892	172,895	161,938	60	56	51
IRI-3	artery clamp	305	137.39	133.52	118.25	143,654	110,275	187,186	52	38	66
IRI-4	artery clamp	357	100.83	103.93	104.08	168,109	84,186	142,952	59	29	50
IRI-5	artery clamp	416	93.60	113.23	97.66	170,834	214,194	315,920	64	74	104
IRI-6	vein clamp	414	94.03	94.90	*missing data**	198,735	103,098	212,018	70	36	70
IRI-7	vein clamp	355	111.24	120.63	114.26	95,008	113,651	212,340	34	39	70
IRI-8	vein clamp	290	94.32	112.96	119.03	343,939	134,740	355,069	117	46	120
IRI-9	vein clamp	291	124.56	124.15	116.22	307,999	145,214	263,802	106	55	89
IRI-10	vein clamp	293	87.01	66.79	58.47	320,375	135,548	174,711	108	45	54
Histology control 1	non	254									
Histology control 2	non	282									
Histology control 3	non	273									
MRI specimen	MRI preparation	345									
Pre-trial rat	non	295									

#IRI = ischemia-reperfusion injury

*unknown error in the blood pressure recording.

Table S2. Image assessment, assignment 1A#

Animal ID*	Assessor 1			Assessor 2			Assessor 3			Assessor 4			Assessor 5			Assessor 6			**Correct**		
	Base-line	Onset	60 min	Base-line	Onset	60 min	Base-line	Onset	60 min	Base-line	Onset	60 min	Base-line	Onset	60 min	Base-line	Onset	60 min	**Base-line**	**Onset**	**60 min**
IRI-9	3	1	2	3	1	2	2	1	3	3	1	2	3	1	2	3	1	2	3	1	2
IRI-3	4	6	5	4	6	5	4	6	5	5	6	4	5	6	4	5	6	4	4	6	5
IRI-6	9	8	7	9	8	7	7	8	9	9	8	7	9	8	7	7	8	9	7	8	9
IRI-8	10	11	12	10	11	12	10	11	12	10	11	12	10	11	12	10	11	12	10	11	12
IRI-5	13	15	14	13	15	14	14	15	13	13	15	14	13	15	14	13	15	14	15	14	13
IRI-1	16	18	17	16	17	18	16	18	17	16	18	17	16	18	17	16	17	18	17	18	16
IRI-7	20	21	19	20	21	19	20	21	19	20	21	19	20	21	19	20	21	19	19	21	20
IRI-10	23	22	24	23	22	24	23	22	24	23	22	24	23	22	24	23	22	24	23	22	24
IRI-4	27	25	26	27	25	26	26	25	27	27	25	26	27	25	26	26	25	27	27	25	26
IRI-2	30	29	28	30	28	29	29	28	30	30	29	28	30	29	28	30	29	28	30	28	29

The six assessors completed assignment 1A and 2A before receiving assignment 1B and 2B.

*Animal ID not known to assessors. Green: Correct answer. Red: Incorrect answer.

The scans from each animal was presented in random order. The scans from the first animal were 1-3, next animal 4-6 etc. The six assessors were presented with the following text: "Below are 10 rat kidneys, each with three super-resolution images in random order. Each kidney was scanned in three different states: 1) baseline (healthy state). 2) at the onset of reperfusion after 45 min of clamping either the renal artery (n=5) or renal vein (n=5) (assumption of low renal blood flow immediately after removal of the clamp). 3) after 60 minutes of reperfusion (assumption that the renal blood flow has returned in some degree). Your task is to assign the images from each rat into three groups based on similarity of the images. Then, you assign the 10 grouped images to either baseline, onset of reperfusion or 60 minutes of reperfusion."

Table S3. Assignment 1A, interobserver agreement

Intervention	True category	Image number	Assessed category		
			Baseline	Onset	60 Min
Vein clamp	Onset	1	0	6	0
	60 Min	2	1	0	5
	Baseline	3	5	0	1
Artery clamp	Baseline	4	3	0	3
	60 Min	5	3	0	3
	Onset	6	0	6	0
Vein clamp	Baseline	7	2	0	4
	Onset	8	0	6	0
	60 Min	9	4	0	2
Vein clamp	Baseline	10	6	0	0
	Onset	11	0	6	0
	60 Min	12	0	0	6
Artery clamp	60 Min	13	5	0	1
	Onset	14	1	0	5
	Baseline	15	0	6	0
Artery clamp	60 Min	16	6	0	0
	Baseline	17	0	2	4
	Onset	18	0	4	2
Vein clamp	Baseline	19	0	0	6
	60 Min	20	6	0	0
	Onset	21	0	6	0
Vein clamp	Onset	22	0	6	0
	Baseline	23	6	0	0
	60 Min	24	0	0	6
Artery clamp	Onset	25	0	6	0
	60 Min	26	2	0	4
	Baseline	27	4	0	2
Artery clamp	Onset	28	0	2	4
	60 Min	29	1	4	1
	Baseline	30	5	0	1

Assessed categories were inserted at the Online Kappa Calculator, available online: http://justusrandolph.net/kappa/ (accessed 27 May 2020).
Results: Percent overall agreement = 73.33%. Fixed-marginal kappa = 0.60.
95% CI for fixed-marginal kappa [0.50, 0.70].

Table S4. Image assessment, assignment 1B

Assessor 1		Assessor 2		Assessor 3		Assessor 4		Assessor 5		Assessor 6		Correct	
Artery	Vein	Artery	Vein	Artery	Vein	Artery	Vein	Artery	Vein	Artery	Vein	**Artery**	**Vein**
A	B	A	B	A	B	A	B	A	B	A	B	A	B
C	D	C	D	C	D	C	D	C	D	C	D	C	E
E	I	G	E	G	E	G	E	F	E	E	G	D	F
H	F	H	F	H	F	H	F	G	I	F	I	G	H
G	J	J	I	J	I	J	I	H	J	H	J	J	I

Image number	Animal ID
A	IRI-5
B	IRI-6
C	IRI-1
D	IRI-4
E	IRI-7
F	IRI-9
G	IRI-3
H	IRI-8
I	IRI-10
J	IRI-2

Green: Correct answer. Red: Incorrect answer. The scans from each animal was presented in the correct order (baseline – onset of reperfusion – after 60 min of reperfusion).
The animals were identified by a capital letter A-J (as shown in the table to the right).
The six assessors were presented with the following text: "Five of the kidneys below had the renal artery clamped for 45 minutes, and the other five kidneys had the renal vein clamped for 45 minutes. The first image was obtained at baseline, the second was obtained at the onset of reperfusion and the third was obtained after 60 minutes of reperfusion. According to the literature, the vein clamp causes the severest tissue damage. Please group the 10 kidneys (A-J) into the five you believe had the artery clamped, and the five you believe had the vein clamped."

Table S5. Image assessment, assignment 2A

Animal ID*	Image number	Assessor 1		Assessor 2		Assessor 3		Assessor 4		Assessor 5		Assessor 6		**Correct**	
		Baseline	60 min	Baseline	60 min	Baseline	60 min	Baseline	60 min	Baseline	60 min	Baseline	60 min	**Baseline**	**60 min**
IRI-5	A	2	1	2	1	2	1	2	1	2	1	2	1	2	1
IRI-4	B	1	2	1	2	1	2	1	2	1	2	1	2	1	2
IRI-10	C	1	2	1	2	1	2	1	2	1	2	1	2	1	2
IRI-2	D	1	2	1	2	1	2	1	2	1	2	1	2	1	2
IRI-6	E	2	1	2	1	2	1	2	1	2	1	2	1	2	1
IRI-7	F	1	2	1	2	1	2	1	2	1	2	1	2	1	2
IRI-1	G	2	1	2	1	2	1	2	1	2	1	2	1	2	1
IRI-8	H	1	2	1	2	1	2	1	2	1	2	1	2	1	2
IRI-3	I	2	1	2	1	2	1	2	1	2	1	2	1	2	1
IRI-9	J	2	1	2	1	2	1	2	1	2	1	2	1	2	1

*Animal ID not known to assessors. Green: Correct answer. Red: Incorrect answer.
The scans from each animal was presented in the random order. The animals were identified by a capital letter A-J (as shown in the left side of the table).
The six assessors were presented with the following text: "The 10 kidneys were also scanned with power Doppler before and after clamping of the renal artery or renal vein. Please group the 20 scans into the 10 baseline scans and the 10 scans after 60 minutes of reperfusion according to the degree of power doppler signal, based on the following information: According to the literature, the renal blood flow is reduced after ischemia-reperfusion. Please name scans *A1* and *A2* etc."

Table S6. Image assessment, assignment 2B

Assessor 1		Assessor 2		Assessor 3		Assessor 4		Assessor 5		Assessor 6		Correct	
Artery	Vein	Artery	Vein	Artery	Vein	Artery	Vein	Artery	Vein	Artery	Vein	**Artery**	**Vein**
B	A	C	A	C	A	B	A	B	A	A	B	B	A
C	F	D	B	D	B	C	F	C	F	D	C	C	F
D	H	E	F	E	F	D	G	D	H	E	F	D	G
E	I	I	G	I	G	E	H	E	I	I	G	E	H
G	J	J	H	J	H	I	J	G	J	J	H	I	J

Green: Correct answer. Red: Incorrect answer. The scans from each animal was presented in the correct order (baseline – after 60 min of reperfusion).

The animals were identified by a capital letter A-J (as shown in the table to the right).

The six assessors were presented with the following text: "Five of the kidneys below had the renal artery clamped for 45 minutes, and the other five kidneys had the renal vein clamped for 45 minutes. The first image was obtained at baseline, the second was obtained after 60 minutes of reperfusion. According to the literature, the vein clamp causes the severest tissue damage. Please group the 10 kidneys (A-J) into the five you believe had the artery clamped, and the five you believe had the vein clamped. They are not in the same order as assignment 1B. Do not compare the images to the images in assignment 1B."

Image number	Animal ID
A	IRI-10
B	IRI-3
C	IRI-1
D	IRI-2
E	IRI-5
F	IRI-9
G	IRI-8
H	IRI-6
I	IRI-4
J	IRI-7

Table S7. Assessment of hematoxylin and eosin staining

	Assessment	Correct
Vein clamp	A	A
	G	G
	L	L
	E	E
	I	I
	O	
Artery clamp	H	H
	M	M
	J	J
	P	B
	C	O
Sham control	B	D
	D	N
	N	F
	F	K
	K	P
		N

Green: Correct answer. Red: Incorrect answer. Specimens not digitalized.
